# Indications and adverse events of teriparatide: based on FDA adverse event reporting system (FAERS)

**DOI:** 10.3389/fphar.2024.1391356

**Published:** 2024-08-07

**Authors:** Ming-Tao Wen, Jia-Cheng Li, Bo-Wen Lu, Hua-Rong Shao, Pei-Xue Ling, Fei Liu, Gang Li, Di Luo

**Affiliations:** ^1^ The First Clinical Medical School, Shandong University of Traditional Chinese Medicine, Shandong, Jinan, China; ^2^ Orthopaedic, Affiliated Hospital of Shandong University of Traditional Chinese Medicine, Shandong, Jinan, China; ^3^ Shandong Academy of Pharmaceutical Science, Key Laboratory of Biopharmaceuticals, Shandong, Jinan, China

**Keywords:** teriparatide, osteoporosis, FAERS, adverse events, pharmacovigilance

## Abstract

**Background:**

Teriparatide is approved for osteoporosis. Post-marketing surveillance is critical given its widespread use.

**Objective:**

To investigate adverse events (AEs) associated with teriparatide using the FAERS database, compare association strengths for key AEs, and explore potential applications to provide clinical reference.

**Methods:**

FAERS data from 2004 to 2023 were analyzed. Reports where teriparatide was the primary suspect drug were included. Adverse events were mapped to System Organ Classes and Preferred Terms. Disproportionality analysis using ROR, PRR, BCPNN and EBGM algorithms was conducted to detect safety signals.

**Results:**

Out of 107,123 reports with teriparatide as the primary suspect, key AEs identified included pain in extremity (PRR: 4.54), muscle spasms (PRR: 5.11), fractures (PRR range: 17.67–552.95), and increased calcium levels (PRR: 50.73). Teriparatide exhibited a stronger association with increased calcium levels (PRR: 50.73) compared to fractures (PRR range: 17.67–552.95). Notably, only 10.86% of AE reports were submitted by physicians and another 10% by other health professionals. Subset analyses showed a higher consistency of reported AEs from health professionals compared to the general dataset. Off-label uses were noted in conditions such as arthritis (0.57%) and cancer (0.12%). For osteoporosis, main AEs were pain (18.2%), fractures (12.4%), muscle spasms (7.7%), and nausea (6.5%), while glucocorticoid-induced osteoporosis AEs included fractures (24.1%), pain (13.2%), decreased bone density (9.8%), and nausea (5.1%).

**Conclusion:**

Our findings provide real-world safety data on teriparatide, revealing key AEs and their association strengths. The low proportion of reports by healthcare professionals suggests the need for cautious interpretation. Continuous vigilance and further research are imperative to guide teriparatide’s clinical use.

## 1 Introduction

Osteoporosis persists as a notable public health issue, characterized by diminished bone density and integrity, thereby heightening susceptibility to fractures (Khosla and Hofbauer, 2017; Reid and Billington, 2022). With the global population aging, the prevalence of osteoporosis is on the rise. Fragility fractures, a primary sequel of osteoporosis, substantially contribute to morbidity, mortality, and healthcare expenditures (Sànchez-Riera and Wilson, 2017).

A spectrum of pharmacotherapeutic modalities exists for managing osteoporosis, including bisphosphonates, denosumab, raloxifene, calcitonin, and teriparatide (Arceo-Mendoza and Camacho, 2021). Pharmacological interventions predominantly encompass antiresorptive agents, impeding bone resorption, and anabolic medications, stimulating bone formation (Reid, 2020). Teriparatide, an anabolic agent, received approval from the U.S. Food and Drug Administration (FDA) in 2002 for treating osteoporosis in high-risk patients (Hodsman et al., 2005; Blick et al., 2008). Operating as a recombinant human parathyroid hormone fragment (rhPTH1-34), teriparatide fosters osteoblastic activity, thereby facilitating bone formation (Blick et al., 2009). Pivotal clinical trials have underscored teriparatide’s efficacy in augmenting bone mineral density and mitigating fracture occurrence (Lindsay et al., 2016; Oswald et al., 2019; Chiba et al., 2022).

While clinical trials have elucidated common adverse events such as nausea, dizziness, limb pain, and headaches, ongoing post-market surveillance assumes paramount significance owing to teriparatide’s widespread usage (Brixen et al., 2004). The drug label incorporates cautions concerning severe adverse effects like osteosarcoma, hypotension, hypercalcemia, and urolithiasis. Given the intricacies of adverse event reporting and the potential for underreporting in clinical trials, real-world data repositories like the FDA Adverse Event Reporting System (FAERS) assume a pivotal role (Dhodapkar et al., 2022). Harnessing FAERS facilitates researchers in comprehensively analyzing post-approval safety data, offering insights into teriparatide’s indications and associated adverse events in actual clinical scenarios. By delineating teriparatide’s safety profile through FAERS scrutiny, this investigation aims to enlighten and optimize clinical decision-making regarding its utilization, ultimately enhancing patient care and safety in osteoporosis management.

## 2 Methods

### 2.1 Study design and data sources

This retrospective analysis aims to investigate the indications and adverse events linked with teriparatide employing the FAERS. FAERS stands as a pivotal repository for post-marketing surveillance, collating spontaneous reports of adverse events associated with sanctioned medications. It facilitates systematic monitoring and evaluation of drug safety profiles, aiding in discerning potential risks and benefits inherent in pharmaceutical products. Reports forwarded to FAERS emanate from diverse sources, encompassing pharmaceutical manufacturers, healthcare practitioners, and consumers, furnishing a comprehensive dataset for pharmacovigilance scrutiny. In this study, data sourced from FAERS spanning the period from teriparatide’s FDA approval in 2004 until the latest available data were scrutinized, obtained from the publicly accessible FAERS Quarterly Data Extract Files (http://www.fda.gov/Drugs/GuidanceComplianceRegulatoryInformation/Surveillance/AdverseDrugEffects/ucm082193.htm).

### 2.2 Data extraction and processing

In our exploration of teriparatide’s indications and associated adverse events through the FAERS database, our data extraction and processing methods were intricately tailored to address the study’s specific objectives. Teriparatide’s involvement in adverse events underwent meticulous categorization into primary suspect, secondary suspect, concomitant, or interacting categories. Particular attention was given to reports where teriparatide was designated as the primary suspect, suggesting its potential contribution to adverse events.

The coding of adverse events was conducted using Preferred Terms sourced from the Medical Dictionary for Regulatory Activities (MedDRA), followed by mapping to System Organ Classes (SOCs) for comprehensive categorization and analysis.

**FIGURE 1:**
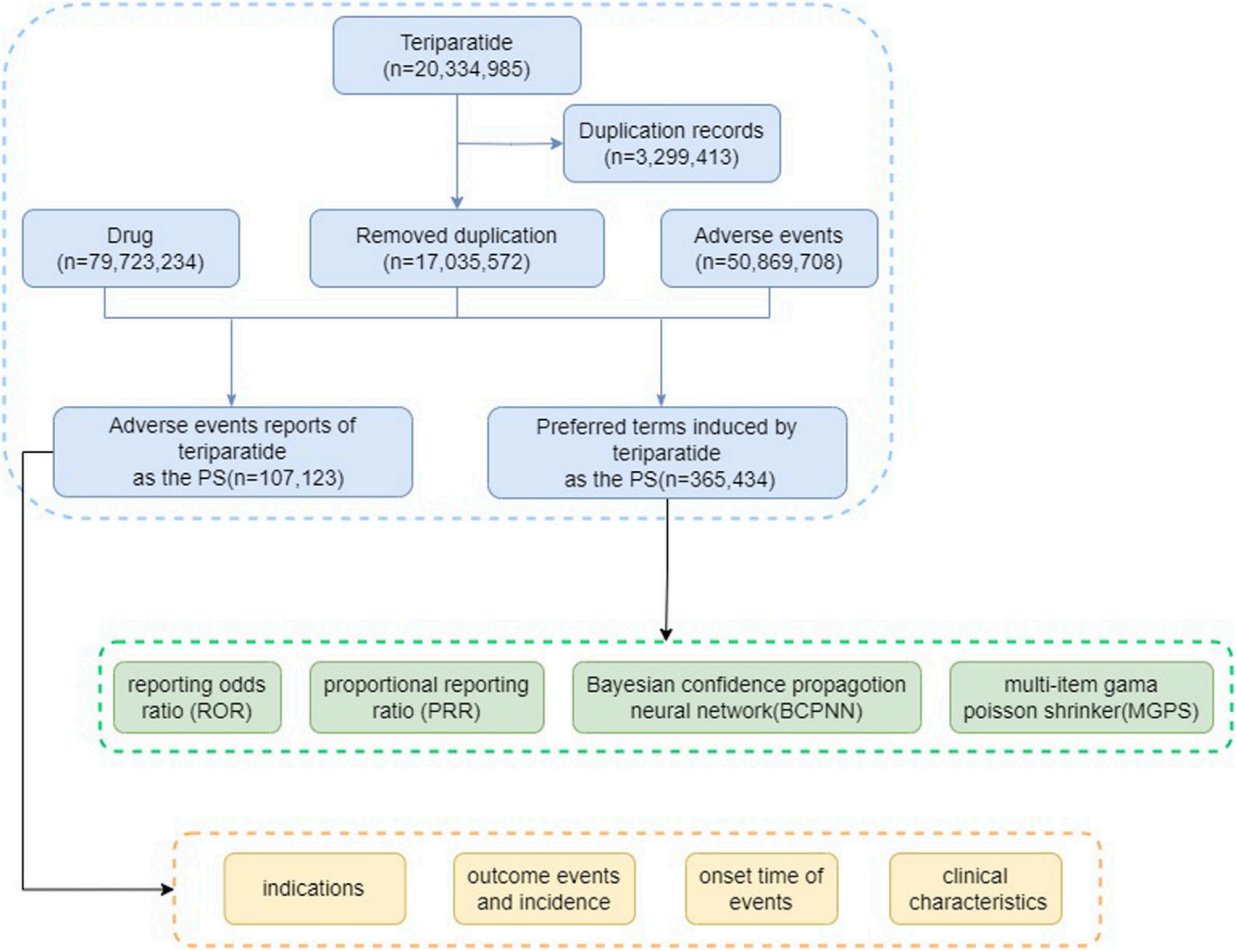
The flow diagram of selecting teriparatide-related AEs from FAERS database.

The visual representation provided in Figure 1 delineates the critical steps involved in our study, including data extraction from FAERS databases, subsequent data processing, deduplication, adverse event classification, summarization of clinical characteristics, and disproportionality analysis between teriparatide and adverse events. Data processing and analysis were conducted utilizing the faersR package (Version 0.0.0.90003) within the R platform (Version 4.3.2), ensuring rigorous and systematic handling of FAERS data. Supplementary data processing tasks were performed using Microsoft Excel 2019 and Graphpad prism 9.5, further reinforcing the accuracy of our analysis.

### 2.3 Data statistics and analysis

Multiple disproportionality analysis techniques were utilized to comprehensively detect safety signals for TP and ABL, including reporting odds ratio (ROR) (Rothman et al., 2004), proportional reporting ratio (PRR) (Evans et al., 2001), Bayesian confidence propagation neural network (BCPNN) (Bate et al., 1998), and multi-item gamma Poisson shrinker (MGPS) (DuMouchel, 1999).

ROR corrects potential bias from small case numbers, while PRR provides higher specificity than ROR. BCPNN enables integrating multi-source data and cross-validation. MGPS is advantageous for detecting rare event signals. Using these complementary methods in combination leverages their respective strengths, allows cross-validation to reduce false positives, and enables detecting more potential rare adverse reactions through threshold and variance adjustments (Jiang et al., 2024).

All algorithms rely on 2 × 2 contingency tables, as illustrated in Table 1. The algorithms, along with their specific formulas and threshold values, are summarized in Table 2. A higher metric value indicates a more pronounced signal, signifying a stronger association between the drug and the adverse events (AEs). By systematically applying these algorithms to mine the FAERS data, this study aimed to identify safety signals for teriparatide comprehensively and reliably. The adoption of a multi-algorithm approach facilitates cross-validation and enhances detection power.

**TABLE 1:** Four grid table.

	Teriparatide-related ADEs	Non-teriparatide-related ADEs	Total
Teriparatide	a	b	a + b
Non-teriparatide	c	d	c + d
Total	a + c	b + d	n = a + b + c + b

**TABLE 2:** Methods, formulas, and thresholds of ROR, PRR, BCPNN, and EBGM.

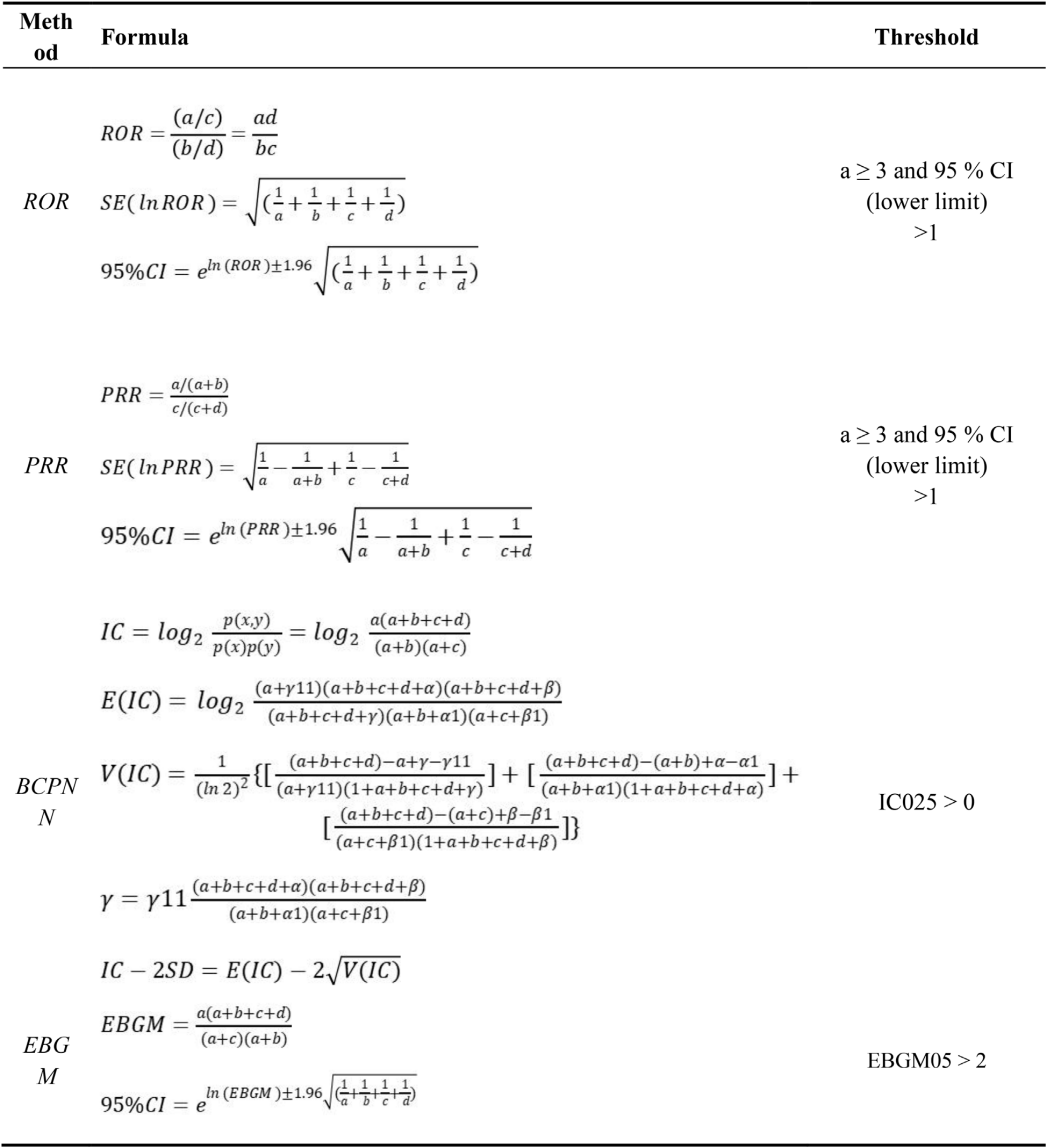

## 3 Results

### 3.1 Descriptive analysis

From 2004 to the third quarter of 2023, this study obtained a total of 17, 035, 572 adverse event reports (AERs) from the FAERS database, including 50, 869, 708 adverse events (AEs). Among these reports, 107,123 identified teriparatide as the primary suspect drug for the AEs. In the AERs involving teriparatide, after excluding cases with unknown age data (43.57%), female patients significantly outnumbered male patients (89.58% vs. 9.26%), and a notable proportion of patients were aged 45 and older (44.79%), consistent with the epidemiology of osteoporosis. The majority of reports (69.81%) originated from consumers rather than healthcare professionals. The United States accounted for the majority of reports (58.94%). In terms of clinical outcomes, apart from unspecified serious AEs, those leading to hospitalization or prolongation of hospitalization were most frequent (47.87%), followed by other serious (38.88%) or death (2.59%). Details can be found in Table 3.

**TABLE 3:** Basic information on AERs related to teriparatide from the FAERS database.

Clinical characteristics	Total number (%)
Year of report	
2004	6,279 (5.86)
2005	4,655 (4.35)
2006	2,134 (1.99)
2007	2,118 (1.98)
2008	2,051 (1.91)
2009	2,926 (2.73)
2010	3,635 (3.39)
2011	5,070 (4.73)
2012	6,138 (5.73)
2013	5,503 (5.14)
2014	1,433 (1.34)
2015	44,728 (41.75)
2016	2,075 (1.94)
2017	3,921 (3.66)
2018	4,734 (4.42)
2019	3,840 (3.58)
2020	1,871 (1.75)
2021	1,601 (1.49)
2022	1,357 (1.27)
2023	1,054 (0.98)
Gender	
female	95,959 (89.58)
Male	9,918 (9.26)
Unkown	1,246 (1.16)
Age	
<18	65 (0.06)
18–45	1,015 (0.95)
45–65	15,294 (14.28)
65–75	17,931 (16.74)
≥75	26,144 (24.41)
Unknow	46,674 (43.57)
Time to event onset (days)	
<7	8,496 (11.21)
7–28	3,275 (4.32)
28–60	2,904 (3.83)
≥60	13,973 (18.44)
Unknow	47,116 (62.19)
Reporter	
Consumer	74,785 (69.81)
Physician	11,638 (10.86)
Unkown	9,285 (8.67)
Other health-professional	6,787 (6.34)
Pharmacist	4,542 (4.24)
Registered Nurse	82 (0.08)
Lawyer	4 (0.00)
Reported countries (top 5)	
United States	63,138 (58.94)
Other	35,009 (32.68)
Spain	2,584 (2.41)
Japan	2,296 (2.14)
Germany	789 (0.74)
Route	
Other	77,404 (72.26)
Subcutaneous	29,550 (27.59)
Oral	102 (0.10)
Intramuscular	53 (0.05)
Intravenous	14 (0.01)
Outcomes	
Hospitalization	24,496 (47.87)
Other serious	19,899 (38.88)
Death	5,460 (10.67)
Disability	641 (1.25)
Life threatening	602 (1.18)
Required intervention to Prevent Permanent Impairment/Damage	72 (0.14)
Congenital anomaly	5 (0.01)
Indications (top 6)	
Osteoporosis	67,224 (62.11)
Senile osteoporosis	1,535 (1.42)
Bone disorder	697 (0.64)
Osteoporotic fracture	683 (0.63)
Osteopenia	424 (0.39)
Osteoporosis postmenopausal	388 (0.36)
Bone density decreased	324 (0.30)

Additionally, a subset analysis of reports from healthcare professionals (Supplementary Table S1) revealed certain differences in year distribution, gender, age, weight, reporter source, reported countries, route of administration, outcomes, and time to event onset compared to the overall situation. For instance, in this subset, the proportion of females was 87.87%, the median age was 74.00 years, the primary reporter was a physician (63.89%), and the proportion of hospitalization outcomes was relatively high (44.17%).

The subset analysis for females (Supplementary Table S2) indicated that all individuals in this subset were female, with a median age of 72.00 years. The majority of reports originated from consumers (68.72%) and the United States (50.63%). The primary route of administration was also categorized as “other” (64.58%), and the proportion of hospitalization outcomes was 44.51%. The main indications in this subset were osteoporosis and decreased bone density.

An analysis of the subset of individuals aged 45 and older (Supplementary Table S3) showed that the proportion of females was 90.58%, with a median age of 72.00 years. The largest number of reports came from the United States, accounting for 58.94% of the total. The primary route of administration was categorized as “other” (69.45%), and the proportion of hospitalization outcomes was 47.42%. Indications in this subset included arthritis, osteoporosis, osteoarthritis, among others.

### 3.2 System organ class

**FIGURE 2:**
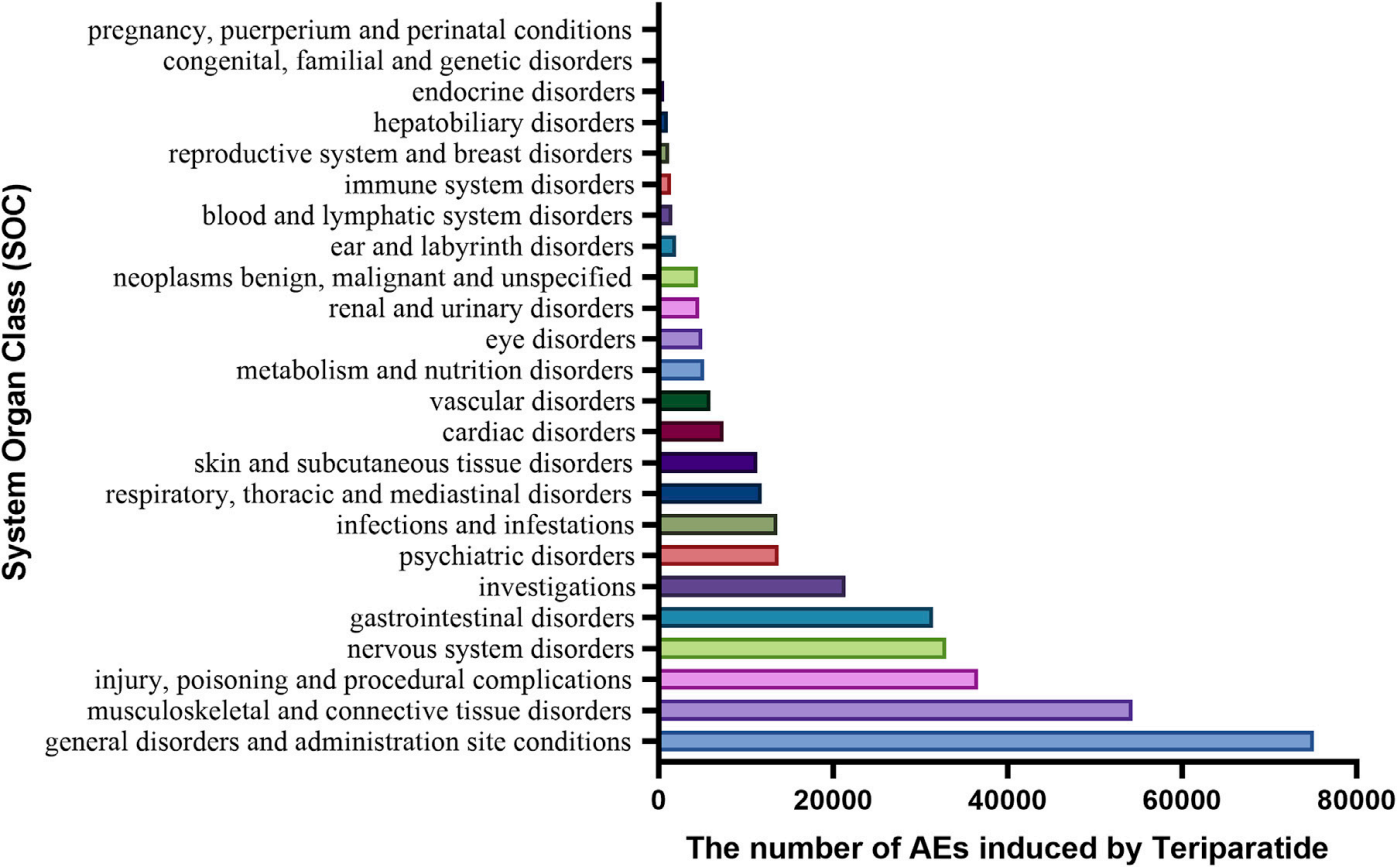
The number of AEs related to teriparatide at the SOC level in FAERS database.

The number of adverse events (AEs) induced by teriparatide as the primary suspect (PS) at the System Organ Class (SOC) level is shown in Figure 2. The study indicates that 3,42,690 adverse event reports (AERs) induced by teriparatide occurred across 24 organ systems. The most common systems were Musculoskeletal and Connective Tissue Disorders (n = 54,348, ROR 3.25, PRR 2.9, IC 1.51, EBGM 2.86) and General Disorders and Administration Site Conditions (n = 75,108, ROR 1.27, PRR 1.21, IC 0.27, EBGM 1.21). Details can be found in Table 4. A subset analysis of reports from healthcare professionals (Supplementary Table S4) shows that at the SOC level, the main signals include Musculoskeletal and Connective Tissue Disorders (ROR 2.41, PRR 2.24, IC 1.16, EBGM 2.24) and Injury, Poisoning and Procedural Complications (ROR 1.75, PRR 1.66, IC 0.73, EBGM 1.65). An analysis of the subset of individuals aged 45 and older (Supplementary Table S5) shows that at the SOC level, the main signals include Musculoskeletal and Connective Tissue Disorders (ROR 3.04, PRR 2.73, IC 1.43, EBGM 2.7) and General Disorders and Administration Site Conditions (ROR 1.39, PRR 1.31, IC 0.39, EBGM 1.31). An analysis of the subset of females (Supplementary Table S6) shows that at the SOC level, the main signals include Musculoskeletal and Connective Tissue Disorders (ROR 3.49, PRR 3.12, IC 1.64, EBGM 3.11) and Injury, Poisoning and Procedural Complications (ROR 1.34, PRR 1.3, IC 0.38, EBGM 1.3).

**TABLE 4:** The signal strength of AERs related to teriparatide at the SOC level in FAERS database detected by four algorithms.

System organ class (SOC)	Case reports	ROR (95% CI)	PRR (95% CI)	χ^2^	IC(IC025)	EBGM(EBGM05)
Musculoskeletal and connective tissue disorders	54,348	3.25 (3.22–3.28)	2.9 (2.9–2.9)	69,983.88	1.51 (1.5)	2.86 (2.84)
General disorders and administration site conditions	75,108	1.27 (1.26–1.28)	1.21 (1.21–1.21)	3,311.58	0.27 (0.26)	1.21 (1.2)
Ear and labyrinth disorders	1951	1.26 (1.21–1.32)	1.26 (1.21–1.31)	105.82	0.33 (0.27)	1.26 (1.21)
Injury, poisoning and procedural complications	36,604	1.17 (1.16–1.18)	1.15 (1.15–1.15)	808.95	0.2 (0.19)	1.15 (1.14)
Nervous system disorders	32,928	1.08 (1.07–1.09)	1.07 (1.05–1.09)	174.62	0.1 (0.08)	1.07 (1.06)
Gastrointestinal disorders	31,450	1.03 (1.02–1.04)	1.02 (1–1.04)	20.75	0.04 (0.02)	1.02 (1.01)
Investigations	21,424	0.95 (0.94–0.97)	0.96 (0.94–0.98)	42.69	−0.06 (−0.08)	0.96 (0.95)
Cardiac disorders	7,413	0.75 (0.74–0.77)	0.76 (0.75–0.78)	588.95	−0.4 (−0.43)	0.76 (0.74)
Vascular disorders	5,892	0.75 (0.73–0.76)	0.75 (0.74–0.76)	502.36	−0.41 (−0.45)	0.75 (0.73)
Infections and infestations	13,578	0.72 (0.7–0.73)	0.73 (0.72–0.74)	1,459.19	−0.46 (−0.48)	0.73 (0.72)
Endocrine disorders	620	0.69 (0.64–0.75)	0.69 (0.64–0.75)	82.88	−0.52 (-0.64)	0.7 (0.65)
Eye disorders	4,981	0.69 (0.67–0.71)	0.7 (0.69–0.71)	669.88	−0.52 (−0.56)	0.7 (0.68)
Renal and urinary disorders	4,592	0.68 (0.66–0.7)	0.69 (0.68–0.7)	674.11	−0.54 (-0.58)	0.69 (0.67)
Respiratory, thoracic and mediastinal disorders	11,756	0.67 (0.66–0.69)	0.69 (0.68–0.7)	1772.3	−0.54 (−0.57)	0.69 (0.68)
Metabolism and nutrition disorders	5,225	0.67 (0.65–0.69)	0.67 (0.66–0.68)	834.87	−0.56 (−0.6)	0.68 (0.66)
Psychiatric disorders	13,704	0.65 (0.64–0.66)	0.66 (0.65–0.67)	2,528.48	−0.59 (−0.62)	0.66 (0.65)
Skin and subcutaneous tissue disorders	11,317	0.58 (0.56–0.59)	0.59 (0.58–0.6)	3,417.95	−0.76 (−0.79)	0.59 (0.58)
Neoplasms benign, malignant and unspecified (incl cysts and polyps)	4,451	0.45 (0.43–0.46)	0.45 (0.44–0.46)	2,996.22	−1.13 (-1.18)	0.46 (0.44)
Reproductive system and breast disorders	1,198	0.39 (0.37–0.42)	0.39 (0.37–0.41)	1,119.74	−1.34 (−1.42)	0.4 (0.38)
Immune system disorders	1,401	0.35 (0.33–0.37)	0.35 (0.33–0.37)	1,667.89	−1.49 (−1.57)	0.36 (0.34)
Hepatobiliary disorders	1,065	0.32 (0.3–0.34)	0.32 (0.3–0.34)	1,508.82	−1.62 (−1.7)	0.33 (0.31)
Blood and lymphatic system disorders	1,560	0.25 (0.24–0.27)	0.26 (0.25–0.28)	3,425.93	−1.96 (−2.03)	0.26 (0.25)
Congenital, familial and genetic disorders	120	0.11 (0.09–0.13)	0.11 (0.09–0.13)	901.73	−3.22 (−3.48)	0.11 (0.09)
Pregnancy, puerperium and perinatal conditions	4	0 (0–0.01)	0 (0–0)	1,584.77	−8.62 (−9.88)	0 (0)

### 3.3 Signal of preferred terms

**FIGURE 3:**
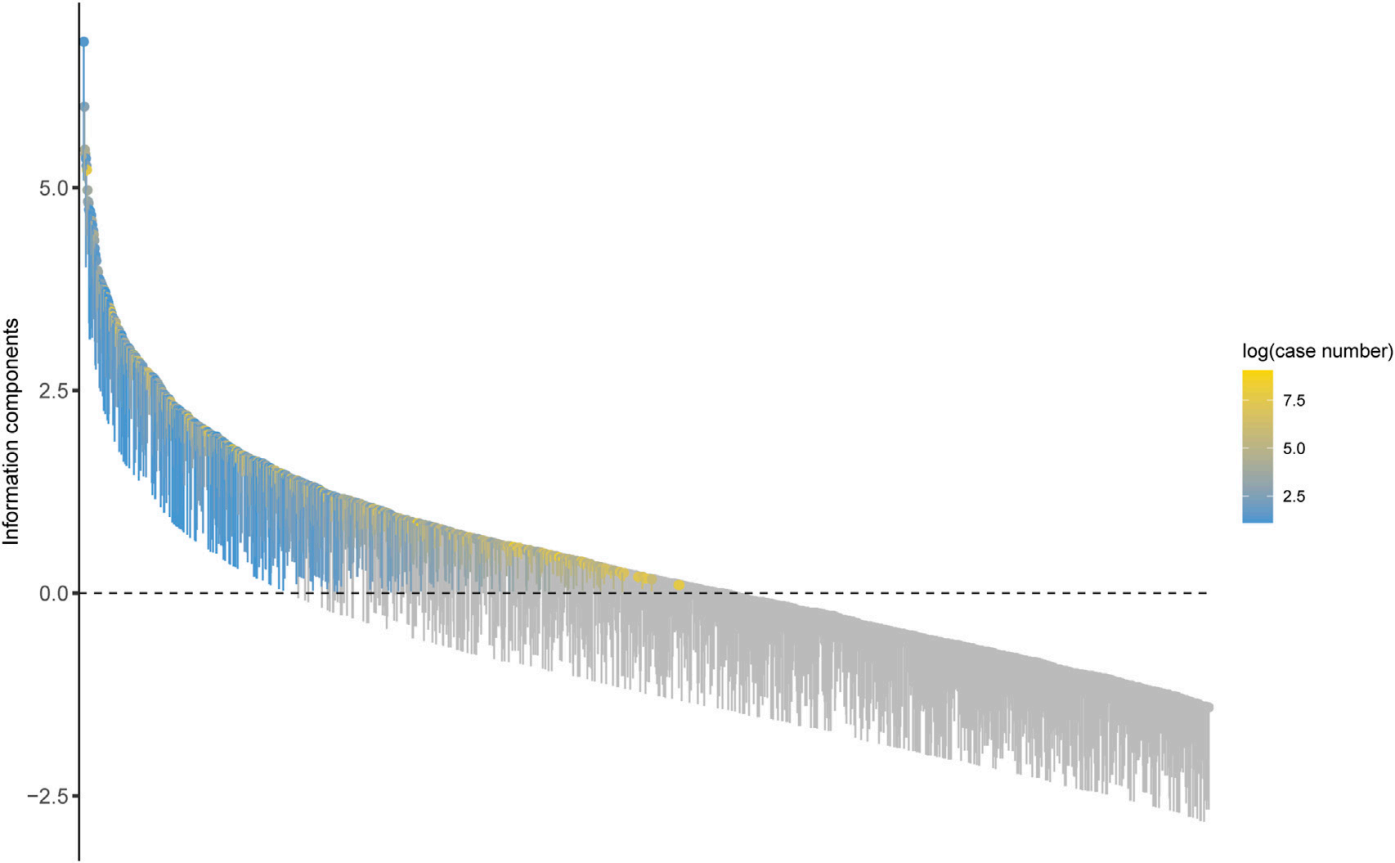
Waterfall plot of all AEs signal intensities of PT level teriparatide in the FAERS database.

At the preferred term (PT) level, our study utilized four algorithms to analyze AERs and evaluate their adherence to various screening criteria, resulting in 318 PTs, as shown in Supplementary Table S1, and the waterfall plot of all AEs signal intensities of PT level is shown in Figure 3. Following ranking based on the EBGM algorithm, we present the top 30 PTs with high signal intensities in Table 5. Among these, we observed significant signal intensities in various injury and musculoskeletal and connective tissue disorders, such as fractures in the limbs or trunk, closely associated with the characteristic of secondary fragility fractures in osteoporosis. Moreover, signals for pain in extremity (n = 8,094, ROR 4.63, PRR 4.54, IC 2.15, EBGM 4.43) and muscle spasms (n = 5,524, ROR 5.18, PRR 5.11, IC 2.27, EBGM 4.96) exhibited notable strength, accompanied by a substantial number of case reports. Furthermore, subset analyses of reports from healthcare professionals (Supplementary Table S7), individuals aged 45 and older (Supplementary Table S8), and females (Supplementary Table S9) revealed higher signal strengths for certain PTs in specific populations, such as osteocalcin increased, growing pains, and urine calcium increased. These findings provide valuable insights into the safety profile of teriparatide across different demographic groups.

**TABLE 5:** The top 30 AEs signal strength of teriparatide at the PTs level in FAERS database detected by four algorithms.

System organ class (SOC)	PTs	Case reports	ROR (95% CI)	PRR (95% CI)	χ^2^	IC(IC025)	EBGM(EBGM05)
Infections and infestations	Urethral carbuncle	4	552.96 (61.8, 4,947.47)	552.95 (61.56, 4,966.48)	440.76	6.8 (5.09)	111.39 (17.8)
Investigations	Osteocalcin increased	17	117.51 (61.55, 224.32)	117.5 (61.54, 224.36)	1,061.45	6 (5.19)	63.97 (37.24)
Musculoskeletal and connective tissue disorders	Growing pains	70	64.52 (48.58, 85.69)	64.51 (49.03, 84.88)	2,984.23	5.47 (5.08)	44.3 (34.94)
Investigations	Urine calcium increased	83	61.05 (47.15, 79.04)	61.03 (47.3, 78.74)	3,399.9	5.41 (5.06)	42.64 (34.36)
Investigations	Blood calcium abnormal	5	57.6 (20.29, 163.5)	57.6 (20.38, 162.77)	196.29	5.36 (4.02)	40.95 (17.11)
Investigations	Urine calcium abnormal	13	52.86 (27.9, 100.16)	52.86 (27.68, 100.93)	478.44	5.27 (4.41)	38.51 (22.56)
Investigations	Blood calcium increased	1988	51.02 (48.46, 53.72)	50.73 (47.83, 53.8)	70,910.4	5.22 (5.15)	37.38 (35.8)
General disorders and administration site conditions	Injection site streaking	44	40.02 (28.61, 55.98)	40.02 (28.68, 55.84)	1,298.06	4.97 (4.5)	31.26 (23.61)
Investigations	Calcium ionised increased	22	35.37 (22.14, 56.49)	35.36 (22.09, 56.6)	584.97	4.83 (4.18)	28.36 (19.17)
General disorders and administration site conditions	Injection site dermatitis	25	34.91 (22.51, 54.14)	34.91 (22.68, 53.73)	657.42	4.81 (4.2)	28.07 (19.45)
Investigations	Procollagen type i c-terminal propeptide increased	4	32.53 (10.94, 96.67)	32.53 (10.85, 97.49)	98.95	4.73 (3.33)	26.52 (10.66)
Investigations	Blood parathyroid hormone	3	31.9 (9.09, 111.95)	31.9 (9.1, 111.83)	72.96	4.71 (3.13)	26.11 (9.13)
Renal and urinary disorders	Bladder malposition acquired	3	31.9 (9.09, 111.95)	31.9 (9.1, 111.83)	72.96	4.71 (3.13)	26.11 (9.13)
Investigations	C-telopeptide increased	11	31.03 (16.14, 59.68)	31.03 (16.25, 59.25)	261.1	4.67 (3.78)	25.53 (14.77)
investigations	scan bone marrow abnormal	4	30.72 (10.4, 90.77)	30.72 (10.45, 90.28)	94.1	4.66 (3.27)	25.32 (10.23)
Musculoskeletal and connective tissue disorders	Osteitis deformans	52	28.99 (21.5, 39.09)	28.99 (21.61, 38.9)	1,161.5	4.59 (4.17)	24.13 (18.79)
Investigations	Serum procollagen type i n-terminal propeptide increased	4	27.65 (9.45, 80.89)	27.65 (9.41, 81.26)	85.61	4.54 (3.15)	23.21 (9.45)
Metabolism and nutrition disorders	Calcium metabolism disorder	26	26.24 (17.25, 39.9)	26.23 (17.38, 39.59)	530.43	4.47 (3.89)	22.21 (15.64)
Investigations	Bone densitometry	7	26.15 (11.66, 58.67)	26.15 (11.71, 58.41)	142.4	4.47 (3.38)	22.15 (11.27)
Investigations	Vitamin d increased	73	25.05 (19.52, 32.14)	25.04 (19.41, 32.31)	1,426.5	4.42 (4.06)	21.35 (17.33)
Musculoskeletal and connective tissue disorders	Bone formation increased	29	23.72 (16, 35.18)	23.72 (16.03, 35.1)	538.7	4.35 (3.8)	20.39 (14.67)
Injury, poisoning and procedural complications	Joint dislocation postoperative	6	21.83 (9.23, 51.63)	21.83 (9.22, 51.71)	102.98	4.25 (3.1)	18.99 (9.24)
Injury, poisoning and procedural complications	Sacroiliac fracture	4	20.48 (7.17, 58.53)	20.48 (7.11, 59.02)	64.55	4.17 (2.81)	17.97 (7.46)
Neoplasms benign, malignant and unspecified (incl cysts and polyps)	Osteoma cutis	4	19.75 (6.93, 56.3)	19.75 (6.99, 55.81)	62.3	4.12 (2.76)	17.4 (7.24)
Investigations	Urine calcium decreased	8	19.4 (9.26, 40.67)	19.4 (9.21, 40.86)	122.44	4.1 (3.09)	17.14 (9.23)
Injury, poisoning and procedural complications	Bone fissure	40	17.72 (12.75, 24.63)	17.72 (12.7, 24.73)	559.45	3.98 (3.52)	15.82 (12.01)
Musculoskeletal and connective tissue disorders	Medial tibial stress syndrome	34	17.67 (12.37, 25.25)	17.67 (12.42, 25.15)	474.09	3.98 (3.48)	15.78 (11.71)
Investigations	Vitamin d abnormal	37	17.46 (12.4, 24.58)	17.46 (12.51, 24.36)	509.66	3.96 (3.48)	15.61 (11.73)
Neoplasms benign, malignant and unspecified (incl cysts and polyps)	Enchondromatosis	7	16.4 (7.49, 35.91)	16.4 (7.49, 35.92)	90.5	3.88 (2.83)	14.77 (7.67)
Psychiatric disorders	Fear of falling	68	16.29 (12.67, 20.95)	16.29 (12.63, 21.02)	873.11	3.88 (3.52)	14.68 (11.9)
Infections and infestations	Urethral carbuncle	4	552.96 (61.8, 4,947.47)	552.95 (61.56, 4,966.48)	440.76	6.8 (5.09)	111.39 (17.8)

In investigations, the most frequent and intense signals were noted for elevated blood calcium levels (n = 1,988, ROR 51.02, PRR 50.73, IC 5.22, EBGM 35.8) and increased heart rate (n = 1,981, ROR 3.41, PRR 3.4, IC 1.74, EBGM 3.34), as previously mentioned in the instructions. However, our study also identified cases of urethral carbuncle (n = 4, ROR 552.96, PRR 552.95, IC 6.8, EBGM 111.39) and acquired bladder malposition (n = 3, ROR 31.9, PRR 31.9, IC 4.71, EBGM 26.11), despite their low number of reports, with exceptionally strong signals.

## 4 Discussion

Studies have shown that subcutaneous teriparatide 20 mg/day is effective in women with postmenopausal osteoporosis, men with idiopathic or hypogonadal osteoporosis and patients with glucocorticoid-induced osteoporosis (Gallagher et al., 2006; Blick et al., 2008; Sethi et al., 2008). And it has been approved by the Food and Drug Administration and the European Medicine Agency for the clinical treatment of osteoporosis and bone destruction caused by glucocorticoids (Minisola et al., 2019; Johnston and Dagar, 2020; Hauser et al., 2021).

The distribution of teriparatide data and the frequency of signals extracted from the FAERS database in this study are consistent with the information provided in the FDA label and instructions. Our comprehensive analysis revealed that the overwhelming majority (89.58%) of AERs associated with teriparatide involved female patients, which should be associated with the higher incidence of postmenopausal osteoporosis in women (Shane et al., 2022). Excluding cases with unknown age data (43.57%), our findings underscored a notable predominance of AEs among patients aged 45 years and older (44.79%), in accordance with the typical epidemiology of osteoporosis. Furthermore, a significant observation emerged regarding the origin of these reports, with the majority emanating from consumers rather than healthcare professionals, indicative of potential underreporting from medical sources, thus emphasizing the imperative for heightened vigilance among healthcare providers in monitoring patients for serious adverse reactions.

The extensive real - world dataset pertaining to teriparatide from FAERS revealed compelling insights. For example, the subset analysis of reports by health - professionals showed that the top signals at the SOC level included musculoskeletal and connective tissue disorders, injury/poisoning/procedural complications, and ear and labyrinth disorders. At the PTs level, notable signals were detected for osteocalcin increased, X-ray of pelvis and hip abnormal, and chondroma. Additionally, the subset analysis of individuals aged 45 and older revealed that the most frequent systems at the SOC level were musculoskeletal and connective tissue disorders, general disorders and administration site conditions, and injury/poisoning/procedural complications. At the PTs level, significant signals were observed for osteocalcin increased, growing pains, and urine calcium increased. These findings not only corroborate teriparatide’s known mechanisms of action but also align with its approved indications for osteoporosis treatment.

Furthermore, employing disproportionality analysis, our study unveiled the potential involvement of various organs or tissues in teriparatide-related adverse events, with gastrointestinal manifestations such as nausea and vomiting emerging as frequent occurrences during teriparatide therapy. Notably, the most prevalent adverse events identified in our study included pain in extremity (n = 8,094), muscle spasms (n = 5,524), fractures, and musculoskeletal pain, consistent with the well-documented effects of teriparatide on bone and muscle physiology. Additionally, the detection of significant signals such as increased blood calcium (n = 1988), elevated heart rate (n = 1981), and heightened urine calcium (n = 83), mirrors the warnings outlined in the teriparatide label. Elevated blood calcium and hypercalcemia or hypercalciuria are relatively common occurrences following teriparatide therapy, resolving immediately upon treatment cessation (Karatoprak et al., 2012; Hajime et al., 2014). Persistent hypercalcemia in patients receiving calcium supplements warrants consideration of teriparatide’s influence (Rossini et al., 2016). Injection site reactions such as streaking and dermatitis were also discerned, aligning with the anticipated effects of subcutaneous drug administration. Of particular significance, our analysis identified disproportionate signals indicative of bone fissure (n = 40), medial tibial stress syndrome (n = 34), enchondromatosis (n = 7), and fear of falling (n = 68), thereby echoing the postmarketing experience of fragility fractures and the risk of falls associated with teriparatide use in real-world settings.

Significantly, although the teriparatide label includes warnings regarding the potential risk of osteosarcoma, our analysis revealed disproportionate signals suggestive of several benign or malignant neoplasms, including osteoma cutis (n = 4) and enchondromatosis (n = 7). Osteosarcoma is the most common non-haematological primary bone malignancy characterised by the production of bone matrix by tumour cells (Rojas et al., 2021). Despite the relatively small case numbers, these findings underscore the importance of sustained vigilance and further investigation into the long-term tumorigenic effects of teriparatide, given its anabolic mechanism of action. While the occurrence of osteosarcoma has not been observed in humans receiving teriparatide treatment at rates different from those in the general population, but in carcinogenicity studies with rats, near lifetime treatment with systemic exposure ranging from 3 to 60 times the exposure in humans was associated with osteosarcoma in rats (Vahle et al., 2002; Vahle et al., 2004; Khan et al., 2017). Therefore, teriparatide’s labeling still includes warnings to physicians and patients about potential tumor risks.

Furthermore, our study unveiled 35 unexpected and significant disproportionate signals that are not currently reported on the teriparatide label, encompassing sensory disturbances (n = 103), growing pains (n = 70), increased waist circumference (n = 21), tinnitus (n = 65), reduced visual acuity (n = 54), urethral carbuncle (n = 4) and acquired bladder malposition (n = 3), among others. The elucidation of the mechanisms and clinical significance underlying these potential associations necessitates meticulous investigation to comprehensively delineate the safety profile of teriparatide in real-world settings.

While our findings largely align with existing safety information, the identification of several unexpected and significant adverse events underscores the imperative for enhanced scrutiny and ongoing research. These revelations serve as a pivotal reference for future investigations and regulatory initiatives aimed at ensuring the safe and efficacious utilization of teriparatide in clinical practice.

## 5 Limitations

This study, employing the FAERS database for teriparatide analysis, effectively addresses the limitations of small clinical study samples and short observation periods, thus providing real-world outcomes post-drug market approval. However, several limitations persist:① FAERS is a voluntary reporting system prone to data bias and underreporting, making it challenging to control confounding factors. Consequently, deeper descriptive and comparative analyses are hindered. Furthermore, the system does not provide total teriparatide user counts, precluding epidemiological analysis and accurate estimation of adverse event occurrence rates post-medication use.② Voluntary reporting extends beyond healthcare professionals, potentially leading to inaccurate drug-ADE associations from non-professional sources.③The PTs often correspond to those already noted in the drug label, diluting signals for off-label and rare ADEs, constraining the assessment of new signal-drug associations.④The statistical methods employed in this study, though sensitive, may exhibit increased false positive rates with rising report numbers.

Additionally, the signals unearthed only indicate a statistical association between the drug and ADEs, and do not necessarily imply a causal relationship between the two. Therefore, further clinical trials are needed to validate and guide clinical medication.

## 6 Conclusion

In conclusion, our comprehensive analysis of FAERS data provides valuable insights into the indications and AEs of teriparatide. Our study shows that reports submitted by healthcare professionals are still underreported compared to consumers, highlighting the importance of healthcare providers being vigilant in monitoring patients for serious adverse reactions. We found unexpected and significant adverse events, including urethral carbuncle and acquired bladder malposition. These findings highlight the need for healthcare providers to adjust their drug choices and closely monitor patients receiving teriparatide.

Furthermore, our study has unearthed numerous postmarketing safety signals congruent with clinical trials, alongside reports necessitating further regulatory scrutiny to ascertain their significance. To optimize teriparatide’s therapeutic utility, forthcoming inquiries are requisite to thoroughly elucidate its safety profile. These findings serve as a pivotal reference for forthcoming in-depth investigations and safety regulatory initiatives, underscoring the perpetual need for vigilant monitoring and research endeavors to uphold the safe and efficacious deployment of teriparatide in clinical settings.

## Data Availability

The datasets presented in this study can be found in online repositories. The names of the repository/repositories and accession number(s) can be found below: http://www.fda.gov/Drugs/GuidanceComplianceRegulatoryInformation/Surveillance/AdverseDrugEffects/ucm082193.htm.
