# Supplementary material for: Indications and adverse events of teriparatide: based on FDA adverse event reporting system (FAERS)
Source: Front Pharmacol. 2024 Aug 7;15:1391356. doi: 10.3389/fphar.2024.1391356 (PMC11335658; doi:10.3389/fphar.2024.1391356)
Supplement: Supplementary file 1 [file Table8.DOCX]

**Table S8** The top 30 AEs signal strength of teriparatide in patients ≥45 at the PTs level in FAERS database detected by four algorithms

| **System organ class**  **(SOC)** | **PTs** | **Case Reports** | **ROR(95% CI)** | **PRR(95% CI)** | **χ^2^** | **IC(IC025)** | **EBGM(EBGM05)** |
| --- | --- | --- | --- | --- | --- | --- | --- |
| investigations | osteocalcin increased | 10 | 109.17(47.87, 248.98) | 109.17(47.93, 248.66) | 605.78 | 5.96(4.93) | 62.14(31.17) |
| musculoskeletal and connective tissue disorders | growing pains | 41 | 69.28(47.69, 100.65) | 69.27(47.73, 100.53) | 1853.81 | 5.55(5.05) | 46.88(34.3) |
| investigations | urine calcium increased | 55 | 66.73(48.43, 91.94) | 66.71(48.75, 91.28) | 2421.68 | 5.51(5.08) | 45.7(34.95) |
| investigations | blood calcium increased | 1241 | 50.95(47.75, 54.37) | 50.67(47.78, 53.74) | 44531.31 | 5.23(5.14) | 37.6(35.61) |
| general disorders and administration site conditions | injection site dermatitis | 21 | 49.68(30.22, 81.65) | 49.67(30.43, 81.08) | 741.87 | 5.21(4.53) | 37.05(24.45) |
| investigations | urine calcium | 7 | 47.31(20.11, 111.28) | 47.31(19.97, 112.07) | 237.97 | 5.16(4.03) | 35.73(17.47) |
| investigations | blood parathyroid hormone | 3 | 47.31(12.81, 174.74) | 47.31(12.72, 175.9) | 101.99 | 5.16(3.54) | 35.73(11.97) |
| renal and urinary disorders | bladder malposition acquired | 3 | 47.31(12.81, 174.74) | 47.31(12.72, 175.9) | 101.99 | 5.16(3.54) | 35.73(11.97) |
| investigations | blood calcium | 3 | 42.58(11.72, 154.71) | 42.58(11.68, 155.24) | 93.69 | 5.04(3.44) | 32.98(11.2) |
| investigations | scan bone marrow abnormal | 4 | 40.55(13.35, 123.19) | 40.55(13.27, 123.93) | 120 | 4.99(3.57) | 31.76(12.53) |
| general disorders and administration site conditions | injection site streaking | 26 | 40.11(25.95, 61.99) | 40.11(26.06, 61.73) | 772.98 | 4.98(4.37) | 31.49(21.88) |
| investigations | c-telopeptide increased | 7 | 39.74(17.19, 91.88) | 39.74(17.11, 92.31) | 206.51 | 4.97(3.85) | 31.26(15.5) |
| investigations | calcium ionised increased | 19 | 34.57(20.94, 57.08) | 34.57(20.77, 57.55) | 498.05 | 4.81(4.11) | 27.99(18.4) |
| investigations | urine calcium decreased | 8 | 29.88(13.94, 64.04) | 29.88(13.91, 64.17) | 184.45 | 4.64(3.61) | 24.86(13.13) |
| metabolism and nutrition disorders | calcium metabolism disorder | 16 | 28.74(16.8, 49.2) | 28.74(16.93, 48.79) | 356.28 | 4.59(3.85) | 24.07(15.35) |
| investigations | mammogram | 3 | 28.38(8.22, 98.05) | 28.38(8.26, 97.56) | 66.05 | 4.57(3.02) | 23.82(8.44) |
| investigations | vitamin d increased | 48 | 27.04(19.86, 36.82) | 27.03(19.75, 36.99) | 1010.79 | 4.52(4.08) | 22.87(17.66) |
| investigations | serum procollagen type i n-terminal propeptide increased | 3 | 26.61(7.75, 91.33) | 26.61(7.74, 91.48) | 62.27 | 4.5(2.95) | 22.57(8.04) |
| investigations | bone densitometry | 4 | 25.8(8.89, 74.88) | 25.8(8.95, 74.35) | 80.7 | 4.46(3.08) | 21.99(9.02) |
| neoplasms benign, malignant and unspecified (incl cysts and polyps) | chondroma | 4 | 24.68(8.54, 71.37) | 24.68(8.56, 71.12) | 77.42 | 4.4(3.03) | 21.17(8.71) |
| musculoskeletal and connective tissue disorders | bone formation increased | 18 | 24.33(14.76, 40.12) | 24.33(14.62, 40.5) | 343.73 | 4.39(3.69) | 20.91(13.76) |
| musculoskeletal and connective tissue disorders | osteitis deformans | 26 | 23.21(15.33, 35.14) | 23.21(15.38, 35.03) | 474.85 | 4.33(3.75) | 20.09(14.2) |
| neoplasms benign, malignant and unspecified (incl cysts and polyps) | enchondromatosis | 5 | 22.18(8.64, 56.91) | 22.17(8.65, 56.8) | 87.44 | 4.27(3.03) | 19.31(8.78) |
| vascular disorders | vessel perforation | 10 | 21.5(11.06, 41.82) | 21.5(11.04, 41.87) | 169.77 | 4.23(3.32) | 18.8(10.78) |
| gastrointestinal disorders | femoral hernia | 7 | 20.7(9.37, 45.74) | 20.7(9.45, 45.34) | 114.51 | 4.19(3.12) | 18.19(9.37) |
| neoplasms benign, malignant and unspecified (incl cysts and polyps) | osteoma cutis | 3 | 20.27(6.05, 67.97) | 20.27(6.01, 68.33) | 48.1 | 4.16(2.64) | 17.86(6.49) |
| investigations | osteocalcin decreased | 3 | 19.35(5.79, 64.66) | 19.35(5.74, 65.23) | 45.95 | 4.1(2.58) | 17.15(6.25) |
| injury, poisoning and procedural complications | wrong product stored | 3 | 17.03(5.14, 56.41) | 17.03(5.15, 56.29) | 40.42 | 3.94(2.43) | 15.31(5.62) |
| investigations | vitamin d abnormal | 21 | 16.84(10.71, 26.47) | 16.84(10.73, 26.43) | 279.66 | 3.92(3.29) | 15.16(10.38) |
| injury, poisoning and procedural complications | joint dislocation postoperative | 3 | 16.38(4.96, 54.1) | 16.38(4.96, 54.14) | 38.83 | 3.89(2.38) | 14.78(5.44) |
| psychiatric disorders | sleep disorder due to general medical condition, hypersomnia type | 3 | 15.21(4.62, 50.02) | 15.21(4.6, 50.28) | 35.96 | 3.79(2.29) | 13.83(5.11) |
| injury, poisoning and procedural complications | compression fracture | 222 | 14.75(12.85, 16.94) | 14.74(12.85, 16.91) | 2575.28 | 3.75(3.55) | 13.44(11.98) |
