# Supplementary material for: Indications and adverse events of teriparatide: based on FDA adverse event reporting system (FAERS)
Source: Front Pharmacol. 2024 Aug 7;15:1391356. doi: 10.3389/fphar.2024.1391356 (PMC11335658; doi:10.3389/fphar.2024.1391356)
Supplement: Supplementary file 2 [file Table7.DOCX]

**Table S7** The top 30 AEs signal strength of teriparatide reported by health-professional at the PTs level in FAERS database detected by four algorithms

| **System organ class**  **(SOC)** | **PTs** | **Case Reports** | **ROR(95% CI)** | **PRR(95% CI)** | **χ^2^** | **IC(IC025)** | **EBGM(EBGM05)** |
| --- | --- | --- | --- | --- | --- | --- | --- |
| investigations | osteocalcin increased | 6 | 108.8(42.89, 275.95) | 108.78(43.3, 273.29) | 473.6 | 6.33(5.12) | 80.67(37.02) |
| musculoskeletal and connective tissue disorders | osteitis deformans | 25 | 72.73(47.03, 112.46) | 72.69(47.23, 111.88) | 1430.35 | 5.88(5.28) | 59.01(40.98) |
| investigations | x-ray of pelvis and hip abnormal | 3 | 54.39(15.94, 185.62) | 54.39(15.82, 186.98) | 133.65 | 5.54(3.99) | 46.38(16.61) |
| neoplasms benign, malignant and unspecified (incl cysts and polyps) | chondroma | 3 | 48.67(14.4, 164.47) | 48.67(14.44, 164.07) | 120.96 | 5.4(3.87) | 42.17(15.22) |
| injury, poisoning and procedural complications | bone fissure | 14 | 46.92(26.74, 82.33) | 46.9(26.57, 82.8) | 545.88 | 5.35(4.57) | 40.84(25.51) |
| investigations | blood calcium increased | 320 | 47.04(41.81, 52.93) | 46.77(41.58, 52.61) | 12445.82 | 5.35(5.18) | 40.74(36.91) |
| neoplasms benign, malignant and unspecified (incl cysts and polyps) | basosquamous carcinoma of skin | 3 | 46.24(13.74, 155.6) | 46.23(13.71, 155.84) | 115.45 | 5.33(3.81) | 40.33(14.61) |
| investigations | calcium ionised increased | 9 | 45.48(22.59, 91.58) | 45.48(22.46, 92.1) | 341.14 | 5.31(4.36) | 39.76(22.13) |
| investigations | urine calcium increased | 12 | 44.04(24.05, 80.64) | 44.03(23.98, 80.84) | 441.57 | 5.27(4.44) | 38.65(23.3) |
| investigations | urine calcium/creatinine ratio increased | 3 | 44.03(13.13, 147.63) | 44.03(13.06, 148.42) | 110.39 | 5.27(3.75) | 38.65(14.05) |
| neoplasms benign, malignant and unspecified (incl cysts and polyps) | enchondromatosis | 4 | 35.23(12.52, 99.12) | 35.23(12.47, 99.55) | 119.37 | 4.99(3.65) | 31.71(13.35) |
| general disorders and administration site conditions | injection site streaking | 5 | 35.03(13.89, 88.34) | 35.03(13.94, 88.01) | 148.4 | 4.98(3.76) | 31.55(14.55) |
| gastrointestinal disorders | femoral hernia | 4 | 32.45(11.58, 90.91) | 32.44(11.48, 91.67) | 110.29 | 4.88(3.54) | 29.45(12.44) |
| musculoskeletal and connective tissue disorders | bone callus excessive | 3 | 31.89(9.71, 104.68) | 31.88(9.64, 105.38) | 81.33 | 4.86(3.36) | 28.99(10.72) |
| musculoskeletal and connective tissue disorders | growing pains | 4 | 30.07(10.77, 83.96) | 30.07(10.85, 83.32) | 102.42 | 4.78(3.45) | 27.49(11.64) |
| injury, poisoning and procedural complications | traumatic shock | 3 | 29.83(9.12, 97.58) | 29.83(9.2, 96.69) | 76.21 | 4.77(3.28) | 27.28(10.12) |
| investigations | urine calcium decreased | 3 | 28.02(8.59, 91.37) | 28.02(8.64, 90.82) | 71.65 | 4.69(3.2) | 25.77(9.58) |
| gastrointestinal disorders | hernial eventration | 5 | 26.12(10.48, 65.09) | 26.12(10.4, 65.62) | 111.36 | 4.59(3.39) | 24.16(11.25) |
| injury, poisoning and procedural complications | pelvic fracture | 154 | 22.93(19.46, 27.01) | 22.86(19.54, 26.74) | 2997.86 | 4.42(4.18) | 21.35(18.62) |
| injury, poisoning and procedural complications | extraskeletal ossification | 6 | 22.56(9.85, 51.67) | 22.55(9.9, 51.36) | 115.16 | 4.4(3.29) | 21.08(10.54) |
| vascular disorders | varicose ulceration | 6 | 22.28(9.73, 51.03) | 22.28(9.78, 50.75) | 113.73 | 4.38(3.27) | 20.85(10.42) |
| neoplasms benign, malignant and unspecified (incl cysts and polyps) | benign bone neoplasm | 4 | 22.02(7.98, 60.72) | 22.02(7.95, 61.02) | 74.9 | 4.37(3.05) | 20.61(8.82) |
| neoplasms benign, malignant and unspecified (incl cysts and polyps) | biliary neoplasm | 3 | 22.02(6.82, 71.03) | 22.02(6.79, 71.38) | 56.17 | 4.37(2.89) | 20.61(7.74) |
| musculoskeletal and connective tissue disorders | fracture malunion | 5 | 21.41(8.65, 52.99) | 21.4(8.69, 52.72) | 90.94 | 4.33(3.13) | 20.08(9.4) |
| gastrointestinal disorders | abdominal strangulated hernia | 5 | 21.11(8.53, 52.25) | 21.11(8.57, 52.01) | 89.65 | 4.31(3.11) | 19.82(9.29) |
| injury, poisoning and procedural complications | wrist fracture | 156 | 19.77(16.81, 23.25) | 19.71(16.85, 23.06) | 2605.1 | 4.22(3.98) | 18.59(16.23) |
| neoplasms benign, malignant and unspecified (incl cysts and polyps) | breast cancer in situ | 27 | 18.79(12.74, 27.72) | 18.79(12.7, 27.81) | 428.54 | 4.15(3.6) | 17.76(12.83) |
| injury, poisoning and procedural complications | hip fracture | 454 | 18.76(17.06, 20.64) | 18.61(16.87, 20.53) | 7139.2 | 4.14(4) | 17.61(16.26) |
| injury, poisoning and procedural complications | patella fracture | 32 | 17.94(12.56, 25.63) | 17.93(12.6, 25.52) | 483.52 | 4.09(3.58) | 17(12.62) |
| renal and urinary disorders | urinary bladder polyp | 6 | 17.78(7.81, 40.5) | 17.78(7.81, 40.5) | 89.85 | 4.08(2.97) | 16.87(8.47) |
