# Supplementary material for: Indications and adverse events of teriparatide: based on FDA adverse event reporting system (FAERS)
Source: Front Pharmacol. 2024 Aug 7;15:1391356. doi: 10.3389/fphar.2024.1391356 (PMC11335658; doi:10.3389/fphar.2024.1391356)
Supplement: Supplementary file 3 [file Table9.DOCX]

**Table S9** The top 30 AEs signal strength of teriparatide in female at the PTs level in FAERS database detected by four algorithms.

| **System organ class**  **(SOC)** | **PTs** | **Case Reports** | **ROR(95% CI)** | **PRR(95% CI)** | **χ^2^** | **IC(IC025)** | **EBGM(EBGM05)** |
| --- | --- | --- | --- | --- | --- | --- | --- |
| investigations | urine calcium/creatinine ratio increased | 3 | 155.87(43.98, 552.39) | 155.85(43.59, 557.18) | 369.26 | 6.96(5.38) | 124.88(43.32) |
| musculoskeletal and connective tissue disorders | bone formation increased | 6 | 93.53(39.65, 220.63) | 93.51(39.48, 221.51) | 477.51 | 6.35(5.2) | 81.44(39.72) |
| investigations | urine calcium increased | 9 | 82.54(41.18, 165.44) | 82.51(41.55, 163.84) | 640 | 6.19(5.24) | 72.98(40.79) |
| musculoskeletal and connective tissue disorders | growing pains | 9 | 82.54(41.18, 165.44) | 82.51(41.55, 163.84) | 640 | 6.19(5.24) | 72.98(40.79) |
| investigations | vitamin d increased | 8 | 64.79(31.28, 134.2) | 64.77(31.36, 133.76) | 455.01 | 5.88(4.89) | 58.77(31.95) |
| musculoskeletal and connective tissue disorders | fracture nonunion | 10 | 55.68(29.16, 106.34) | 55.66(29.15, 106.28) | 492.8 | 5.68(4.79) | 51.18(29.78) |
| musculoskeletal and connective tissue disorders | pseudarthrosis | 4 | 54.22(19.52, 150.63) | 54.21(19.56, 150.21) | 192.2 | 5.64(4.32) | 49.95(21.24) |
| injury, poisoning and procedural complications | extraskeletal ossification | 4 | 41.57(15.11, 114.38) | 41.56(15, 115.16) | 148.44 | 5.29(3.97) | 39.03(16.73) |
| musculoskeletal and connective tissue disorders | fracture pain | 5 | 38.49(15.6, 94.98) | 38.48(15.62, 94.8) | 171.93 | 5.18(3.99) | 36.3(17.05) |
| cardiac disorders | heart valve calcification | 5 | 38.02(15.41, 93.79) | 38.01(15.43, 93.64) | 169.84 | 5.17(3.97) | 35.89(16.86) |
| investigations | blood parathyroid hormone decreased | 14 | 35.35(20.63, 60.58) | 35.33(20.81, 59.98) | 442.04 | 5.07(4.31) | 33.49(21.34) |
| investigations | blood calcium increased | 86 | 34.35(27.63, 42.69) | 34.24(27.6, 42.48) | 2630.6 | 5.02(4.71) | 32.51(27.1) |
| injury, poisoning and procedural complications | compression fracture | 41 | 27.12(19.83, 37.08) | 27.08(19.79, 37.05) | 986.83 | 4.7(4.25) | 25.99(20) |
| injury, poisoning and procedural complications | bone fissure | 3 | 26.72(8.41, 84.87) | 26.72(8.41, 84.93) | 71.21 | 4.68(3.23) | 25.66(9.76) |
| musculoskeletal and connective tissue disorders | bone development abnormal | 4 | 25.71(9.46, 69.9) | 25.71(9.46, 69.86) | 91.22 | 4.63(3.33) | 24.73(10.71) |
| musculoskeletal and connective tissue disorders | osteitis deformans | 3 | 25.62(8.08, 81.3) | 25.62(8.06, 81.43) | 68.17 | 4.62(3.17) | 24.65(9.38) |
| injury, poisoning and procedural complications | fractured sacrum | 4 | 21.5(7.93, 58.26) | 21.5(7.91, 58.42) | 75.57 | 4.38(3.09) | 20.81(9.04) |
| injury, poisoning and procedural complications | spinal fracture | 119 | 19.12(15.93, 22.96) | 19.04(15.96, 22.71) | 1974.38 | 4.21(3.95) | 18.51(15.88) |
| investigations | blood 25-hydroxycholecalciferol decreased | 3 | 18.89(5.99, 59.59) | 18.89(5.94, 60.04) | 49.34 | 4.2(2.76) | 18.36(7.02) |
| renal and urinary disorders | hypercalciuria | 5 | 18.56(7.62, 45.17) | 18.55(7.68, 44.81) | 80.64 | 4.17(3) | 18.05(8.57) |
| musculoskeletal and connective tissue disorders | bone pain | 319 | 18.75(16.77, 20.98) | 18.54(16.48, 20.85) | 5143.23 | 4.17(4.01) | 18.03(16.42) |
| renal and urinary disorders | urinary bladder polyp | 4 | 16.85(6.24, 45.5) | 16.85(6.2, 45.78) | 58.06 | 4.04(2.75) | 16.43(7.16) |
| cardiac disorders | aortic valve calcification | 5 | 16.76(6.89, 40.75) | 16.76(6.94, 40.49) | 72.15 | 4.03(2.86) | 16.35(7.77) |
| psychiatric disorders | fear of falling | 4 | 16.63(6.16, 44.88) | 16.62(6.12, 45.16) | 57.21 | 4.02(2.73) | 16.22(7.07) |
| skin and subcutaneous tissue disorders | needle track marks | 3 | 16.55(5.26, 52.1) | 16.55(5.31, 51.58) | 42.7 | 4.01(2.58) | 16.15(6.19) |
| neoplasms benign, malignant and unspecified (incl cysts and polyps) | osteosarcoma | 4 | 16.41(6.08, 44.28) | 16.41(6.04, 44.59) | 56.38 | 4(2.72) | 16.01(6.98) |
| injury, poisoning and procedural complications | pelvic fracture | 28 | 16.16(11.11, 23.53) | 16.15(11.13, 23.44) | 387.84 | 3.98(3.45) | 15.77(11.52) |
| injury, poisoning and procedural complications | scapula fracture | 4 | 15.3(5.67, 41.26) | 15.3(5.63, 41.57) | 52.18 | 3.9(2.62) | 14.96(6.52) |
| injury, poisoning and procedural complications | lumbar vertebral fracture | 24 | 15.16(10.11, 22.73) | 15.14(10.03, 22.85) | 309.53 | 3.89(3.32) | 14.81(10.55) |
| investigations | blood parathyroid hormone increased | 17 | 15.08(9.32, 24.41) | 15.08(9.24, 24.62) | 218.14 | 3.88(3.21) | 14.74(9.86) |
