# Supplementary material for: Indications and adverse events of teriparatide: based on FDA adverse event reporting system (FAERS)
Source: Front Pharmacol. 2024 Aug 7;15:1391356. doi: 10.3389/fphar.2024.1391356 (PMC11335658; doi:10.3389/fphar.2024.1391356)
Supplement: Supplementary file 4 [file Table2.DOCX]

**Table S2** Basic Information on teriparatide-related AEs in female from the FAERS database.

| **variable** | **Total** |
| --- | --- |
| **Year** |  |
| 2004 | 395( 4.17) |
| 2005 | 287( 3.03) |
| 2006 | 170( 1.79) |
| 2007 | 120( 1.27) |
| 2008 | 186( 1.96) |
| 2009 | 339( 3.58) |
| 2010 | 455( 4.80) |
| 2011 | 580( 6.12) |
| 2012 | 808( 8.53) |
| 2013 | 782( 8.26) |
| 2014 | 162( 1.71) |
| 2015 | 2984(31.51) |
| 2016 | 229( 2.42) |
| 2017 | 383( 4.04) |
| 2018 | 461( 4.87) |
| 2019 | 357( 3.77) |
| 2020 | 223( 2.35) |
| 2021 | 204( 2.15) |
| 2022 | 208( 2.20) |
| 2023 | 138( 1.46) |
| **sex** |  |
| male | 9471(100.00) |
| **age_yr** | 72.00(62.00,80.00) |
| **wt** | 71.21(62.00,82.10) |
| **Reporter** |  |
| Consumer | 6508(68.72) |
| Physician | 1330(14.04) |
| unknown | 611( 6.45) |
| Other health-professional | 587( 6.20) |
| Pharmacist | 431( 4.55) |
| Registered Nurse | 4( 0.04) |
| **Reported countries** |  |
| United States | 4795(50.63) |
| other | 3955(41.76) |
| Spain | 370( 3.91) |
| Japan | 351( 3.71) |
| **route** |  |
| other | 6116(64.58) |
| subcutaneous | 3344(35.31) |
| oral | 11( 0.12) |
| **Outcomes** |  |
| hospitalization | 2823(44.51) |
| other serious | 2100(33.11) |
| death | 1229(19.38) |
| life threatening | 112( 1.77) |
| disability | 71( 1.12) |
| required intervention to Prevent Permanent Impairment/Damage | 7( 0.11) |
| congenital anomaly | 1( 0.02) |
| **tto** | 62.00(6.00,245.00) |
| **ttoQ** |  |
| <7 | 673(10.12) |
| 7~28 | 310( 4.66) |
| 28~60 | 274( 4.12) |
| >=60 | 1343(20.19) |
| unknow | 4052(60.91) |
| **Indications** |  |
| bone density decreased | 44( 0.46) |
| bone disorder | 73( 0.76) |
| compression fracture | 17( 0.18) |
| fracture | 29( 0.30) |
| hypoparathyroidism | 10( 0.10) |
| osteoarthritis | 17( 0.18) |
| osteopenia | 48( 0.50) |
| osteoporosis | 5832(60.97) |
| osteoporotic fracture | 80( 0.84) |
| others | 224( 2.34) |
| pathological fracture | 25( 0.26) |
| product used for unknown indication | 721( 7.54) |
| rheumatoid arthritis | 27( 0.28) |
| senile osteoporosis | 112( 1.17) |
| spinal fracture | 35( 0.37) |
| unknown | 2271(23.74) |
