# Supplementary material for: Indications and adverse events of teriparatide: based on FDA adverse event reporting system (FAERS)
Source: Front Pharmacol. 2024 Aug 7;15:1391356. doi: 10.3389/fphar.2024.1391356 (PMC11335658; doi:10.3389/fphar.2024.1391356)
Supplement: Supplementary file 5 [file Table5.DOCX]

**Table S5** The AEs signal strength of teriparatide in patients ≥45 at SOC Level in FAERS database detected by four algorithms.

| **System organ class**  **(SOC)** | | **Case Reports** | | **ROR(95% CI)** | | **PRR(95% CI)** | | **χ^2^** | **IC(IC025)** | | **EBGM(EBGM05)** | |
| --- | --- | --- | --- | --- | --- | --- | --- | --- | --- | --- | --- | --- |
| musculoskeletal and connective tissue disorders | 32953 | | 3.04(3, 3.08) | | 2.73(2.68, 2.78) | | 37548.64 | | | 1.43(1.41) | | 2.7(2.67) |
| general disorders and administration site conditions | 46963 | | 1.39(1.38, 1.41) | | 1.31(1.31, 1.31) | | 4067.12 | | | 0.39(0.37) | | 1.31(1.29) |
| injury, poisoning and procedural complications | 23574 | | 1.31(1.29, 1.32) | | 1.27(1.25, 1.3) | | 1490.61 | | | 0.34(0.33) | | 1.27(1.26) |
| ear and labyrinth disorders | 1250 | | 1.29(1.22, 1.36) | | 1.29(1.22, 1.37) | | 80.25 | | | 0.36(0.28) | | 1.29(1.23) |
| nervous system disorders | 21451 | | 1.08(1.07, 1.1) | | 1.07(1.05, 1.09) | | 115.18 | | | 0.1(0.08) | | 1.07(1.06) |
| gastrointestinal disorders | 20113 | | 0.99(0.98, 1.01) | | 0.99(0.97, 1.01) | | 0.68 | | | -0.01(-0.03) | | 0.99(0.98) |
| investigations | 13751 | | 0.91(0.9, 0.93) | | 0.92(0.9, 0.94) | | 110.82 | | | -0.12(-0.15) | | 0.92(0.9) |
| vascular disorders | 3884 | | 0.74(0.72, 0.76) | | 0.75(0.72, 0.78) | | 343.53 | | | -0.42(-0.47) | | 0.75(0.73) |
| cardiac disorders | 4902 | | 0.72(0.7, 0.74) | | 0.72(0.71, 0.73) | | 537.01 | | | -0.47(-0.51) | | 0.72(0.71) |
| eye disorders | 3045 | | 0.7(0.68, 0.73) | | 0.71(0.68, 0.74) | | 379.57 | | | -0.5(-0.55) | | 0.71(0.69) |
| infections and infestations | 8738 | | 0.69(0.67, 0.7) | | 0.7(0.69, 0.71) | | 1197.17 | | | -0.51(-0.54) | | 0.7(0.69) |
| psychiatric disorders | 8545 | | 0.68(0.66, 0.69) | | 0.69(0.68, 0.7) | | 1267.67 | | | -0.53(-0.57) | | 0.69(0.68) |
| renal and urinary disorders | 2930 | | 0.67(0.64, 0.69) | | 0.67(0.64, 0.7) | | 475.63 | | | -0.57(-0.62) | | 0.67(0.65) |
| respiratory, thoracic and mediastinal disorders | 7544 | | 0.63(0.62, 0.64) | | 0.64(0.63, 0.65) | | 1577.56 | | | -0.63(-0.67) | | 0.64(0.63) |
| metabolism and nutrition disorders | 3341 | | 0.62(0.6, 0.64) | | 0.62(0.6, 0.64) | | 768.55 | | | -0.67(-0.72) | | 0.63(0.61) |
| endocrine disorders | 355 | | 0.59(0.53, 0.66) | | 0.59(0.53, 0.65) | | 98.18 | | | -0.75(-0.9) | | 0.6(0.55) |
| skin and subcutaneous tissue disorders | 6930 | | 0.55(0.54, 0.56) | | 0.56(0.55, 0.57) | | 2469.03 | | | -0.82(-0.86) | | 0.57(0.55) |
| neoplasms benign, malignant and unspecified (incl cysts and polyps) | 2657 | | 0.45(0.44, 0.47) | | 0.46(0.44, 0.48) | | 1728.27 | | | -1.12(-1.17) | | 0.46(0.45) |
| reproductive system and breast disorders | 791 | | 0.43(0.4, 0.46) | | 0.43(0.4, 0.47) | | 585.47 | | | -1.2(-1.3) | | 0.44(0.41) |
| immune system disorders | 840 | | 0.35(0.33, 0.37) | | 0.35(0.33, 0.37) | | 1011.9 | | | -1.5(-1.6) | | 0.35(0.33) |
| hepatobiliary disorders | 672 | | 0.28(0.26, 0.3) | | 0.28(0.26, 0.3) | | 1271.88 | | | -1.84(-1.95) | | 0.28(0.26) |
| blood and lymphatic system disorders | 996 | | 0.22(0.21, 0.24) | | 0.23(0.22, 0.24) | | 2657.57 | | | -2.13(-2.22) | | 0.23(0.22) |
| congenital, familial and genetic disorders | 70 | | 0.15(0.12, 0.19) | | 0.15(0.12, 0.19) | | 336.93 | | | -2.73(-3.06) | | 0.15(0.12) |
