# Supplementary material for: Indications and adverse events of teriparatide: based on FDA adverse event reporting system (FAERS)
Source: Front Pharmacol. 2024 Aug 7;15:1391356. doi: 10.3389/fphar.2024.1391356 (PMC11335658; doi:10.3389/fphar.2024.1391356)
Supplement: Supplementary file 6 [file Table3.DOCX]

**Table S3** Basic Information on teriparatide-related AEs in individuals aged 45 and older from the FAERS database.

| **variable** | **Total** |
| --- | --- |
| **Year** |  |
| 2004 | 5355( 9.15) |
| 2005 | 4147( 7.09) |
| 2006 | 1711( 2.93) |
| 2007 | 1536( 2.63) |
| 2008 | 1063( 1.82) |
| 2009 | 1387( 2.37) |
| 2010 | 2195( 3.75) |
| 2011 | 3284( 5.61) |
| 2012 | 3403( 5.82) |
| 2013 | 2237( 3.82) |
| 2014 | 838( 1.43) |
| 2015 | 21327(36.46) |
| 2016 | 1085( 1.85) |
| 2017 | 1577( 2.70) |
| 2018 | 2062( 3.53) |
| 2019 | 2352( 4.02) |
| 2020 | 993( 1.70) |
| 2021 | 826( 1.41) |
| 2022 | 633( 1.08) |
| 2023 | 484( 0.83) |
| **sex** |  |
| female | 52985(90.58) |
| male | 5234( 8.95) |
| unknown | 276( 0.47) |
| **age_yr** | 72.00(64.00,80.00) |
| **wt** | 59.00(51.00,70.00) |
| **Reporter** |  |
| Consumer | 37124(63.47) |
| unknown | 8035(13.74) |
| Physician | 7479(12.79) |
| Pharmacist | 3056( 5.22) |
| Other health-professional | 2742( 4.69) |
| Registered Nurse | 56( 0.10) |
| Lawyer | 3( 0.01) |
| **Reported countries** |  |
| United States | 29747(50.85) |
| other | 24735(42.29) |
| Japan | 1193( 2.04) |
| Taiwan | 621( 1.06) |
| Spain | 476( 0.81) |
| France | 425( 0.73) |
| Germany | 370( 0.63) |
| Canada | 361( 0.62) |
| Italy | 334( 0.57) |
| Brazil | 233( 0.40) |
| **route** |  |
| other | 40626(69.45) |
| subcutaneous | 17757(30.36) |
| oral | 63( 0.11) |
| intramuscular | 39( 0.07) |
| intravenous | 10( 0.02) |
| **Outcomes** |  |
| hospitalization | 14629(47.42) |
| other serious | 12092(39.19) |
| death | 3236(10.49) |
| disability | 435( 1.41) |
| life threatening | 408( 1.32) |
| required intervention to Prevent Permanent Impairment/Damage | 47( 0.15) |
| congenital anomaly | 4( 0.01) |
| **tto** | 52.00(3.00,214.00) |
| **ttoQ** |  |
| <7 | 7090(15.04) |
| 7~28 | 2848( 6.04) |
| 28~60 | 2451( 5.20) |
| >=60 | 11527(24.45) |
| unknow | 23222(49.26) |
| **Indications** |  |
| arthritis | 20( 0.03) |
| arthropathy | 16( 0.03) |
| back disorder | 10( 0.02) |
| back pain | 14( 0.02) |
| bone density abnormal | 48( 0.08) |
| bone density decreased | 247( 0.42) |
| bone disorder | 403( 0.68) |
| bone loss | 22( 0.04) |
| compression fracture | 53( 0.09) |
| fall | 13( 0.02) |
| femur fracture | 26( 0.04) |
| fracture | 150( 0.25) |
| gastrooesophageal reflux disease | 12( 0.02) |
| hip fracture | 29( 0.05) |
| humerus fracture | 11( 0.02) |
| hypocalcaemia | 18( 0.03) |
| hypoparathyroidism | 60( 0.10) |
| ill-defined disorder | 11( 0.02) |
| lumbar vertebral fracture | 13( 0.02) |
| multiple fractures | 59( 0.10) |
| osteoarthritis | 123( 0.21) |
| osteogenesis imperfecta | 28( 0.05) |
| osteomalacia | 11( 0.02) |
| osteopenia | 264( 0.45) |
| osteoporosis | 41350(70.05) |
| osteoporosis postmenopausal | 242( 0.41) |
| osteoporotic fracture | 382( 0.65) |
| others | 480( 0.81) |
| pathological fracture | 91( 0.15) |
| pelvic fracture | 22( 0.04) |
| product used for unknown indication | 2102( 3.56) |
| prophylaxis | 27( 0.05) |
| psoriasis | 16( 0.03) |
| rheumatoid arthritis | 125( 0.21) |
| rib fracture | 13( 0.02) |
| senile osteoporosis | 859( 1.46) |
| spinal compression fracture | 47( 0.08) |
| spinal disorder | 20( 0.03) |
| spinal fracture | 161( 0.27) |
| stress fracture | 26( 0.04) |
| unknown | 11405(19.32) |
