# Supplementary material for: Indications and adverse events of teriparatide: based on FDA adverse event reporting system (FAERS)
Source: Front Pharmacol. 2024 Aug 7;15:1391356. doi: 10.3389/fphar.2024.1391356 (PMC11335658; doi:10.3389/fphar.2024.1391356)
Supplement: Supplementary file 8 [file Table4.DOCX]

**Table S4** The AEs signal strength of teriparatide reported by health-professional at SOC level in FAERS database detected by four algorithms.

| **System organ class**  **(SOC)** | **Case Reports** | **ROR(95% CI)** | **PRR(95% CI)** | **χ^2^** | **IC(IC025)** | **EBGM(EBGM05)** |
| --- | --- | --- | --- | --- | --- | --- |
| musculoskeletal and connective tissue disorders | 6136 | 2.41(2.34, 2.47) | 2.24(2.2, 2.28) | 4433.13 | 1.16(1.12) | 2.24(2.19) |
| injury, poisoning and procedural complications | 6834 | 1.75(1.71, 1.8) | 1.66(1.63, 1.69) | 1920.45 | 0.73(0.69) | 1.65(1.62) |
| ear and labyrinth disorders | 283 | 1.41(1.25, 1.59) | 1.41(1.25, 1.59) | 33.43 | 0.49(0.32) | 1.41(1.27) |
| nervous system disorders | 5215 | 1.14(1.11, 1.18) | 1.13(1.11, 1.15) | 85.98 | 0.18(0.14) | 1.13(1.1) |
| cardiac disorders | 2252 | 1.1(1.06, 1.15) | 1.1(1.06, 1.14) | 20.44 | 0.13(0.07) | 1.1(1.06) |
| general disorders and administration site conditions | 8717 | 1.05(1.02, 1.07) | 1.04(1.02, 1.06) | 16.24 | 0.06(0.02) | 1.04(1.02) |
| investigations | 3799 | 0.98(0.95, 1.01) | 0.98(0.94, 1.02) | 1.22 | -0.02(-0.07) | 0.98(0.96) |
| gastrointestinal disorders | 4450 | 0.97(0.94, 1) | 0.97(0.95, 0.99) | 3.76 | -0.04(-0.08) | 0.97(0.95) |
| metabolism and nutrition disorders | 1370 | 0.95(0.9, 1) | 0.95(0.9, 1.01) | 3.97 | -0.08(-0.15) | 0.95(0.91) |
| vascular disorders | 1290 | 0.94(0.89, 0.99) | 0.94(0.89, 1) | 4.67 | -0.09(-0.17) | 0.94(0.9) |
| infections and infestations | 3062 | 0.89(0.86, 0.92) | 0.89(0.86, 0.93) | 41.12 | -0.16(-0.21) | 0.89(0.87) |
| endocrine disorders | 162 | 0.88(0.76, 1.03) | 0.88(0.75, 1.03) | 2.51 | -0.18(-0.4) | 0.88(0.78) |
| neoplasms benign, malignant and unspecified (incl cysts and polyps) | 1520 | 0.84(0.8, 0.89) | 0.85(0.8, 0.9) | 42.26 | -0.24(-0.31) | 0.85(0.81) |
| renal and urinary disorders | 908 | 0.81(0.76, 0.87) | 0.82(0.77, 0.87) | 38.71 | -0.29(-0.39) | 0.82(0.77) |
| respiratory, thoracic and mediastinal disorders | 2366 | 0.8(0.77, 0.83) | 0.81(0.78, 0.84) | 112.41 | -0.3(-0.36) | 0.81(0.78) |
| eye disorders | 691 | 0.66(0.61, 0.71) | 0.66(0.61, 0.71) | 120.8 | -0.59(-0.7) | 0.66(0.62) |
| reproductive system and breast disorders | 228 | 0.65(0.57, 0.74) | 0.65(0.57, 0.75) | 41.75 | -0.61(-0.8) | 0.66(0.59) |
| skin and subcutaneous tissue disorders | 1534 | 0.55(0.53, 0.58) | 0.57(0.54, 0.6) | 530.93 | -0.81(-0.89) | 0.57(0.54) |
| psychiatric disorders | 1536 | 0.55(0.52, 0.58) | 0.56(0.53, 0.59) | 552.52 | -0.83(-0.9) | 0.56(0.54) |
| hepatobiliary disorders | 339 | 0.41(0.37, 0.46) | 0.42(0.38, 0.46) | 283.27 | -1.27(-1.42) | 0.42(0.38) |
| immune system disorders | 234 | 0.3(0.27, 0.34) | 0.31(0.27, 0.36) | 373.15 | -1.7(-1.89) | 0.31(0.28) |
| blood and lymphatic system disorders | 436 | 0.27(0.24, 0.3) | 0.27(0.24, 0.3) | 859.17 | -1.86(-2) | 0.28(0.25) |
| congenital, familial and genetic disorders | 41 | 0.16(0.12, 0.22) | 0.16(0.12, 0.22) | 175.4 | -2.6(-3.04) | 0.16(0.13) |
