# Supplementary material for: Indications and adverse events of teriparatide: based on FDA adverse event reporting system (FAERS)
Source: Front Pharmacol. 2024 Aug 7;15:1391356. doi: 10.3389/fphar.2024.1391356 (PMC11335658; doi:10.3389/fphar.2024.1391356)
Supplement: Supplementary file 9 [file Table6.DOCX]

**Table S6** The AEs signal strength of teriparatide in female at SOC Level in FAERS database detected by four algorithms.

| **System organ class**  **(SOC)** | **Case Reports** | **ROR(95% CI)** | **PRR(95% CI)** | **χ^2^** | **IC(IC025)** | **EBGM(EBGM05)** |
| --- | --- | --- | --- | --- | --- | --- |
| musculoskeletal and connective tissue disorders | 3806 | 3.49(3.37, 3.61) | 3.12(3, 3.24) | 5739.75 | 1.64(1.59) | 3.11(3.03) |
| injury, poisoning and procedural complications | 2949 | 1.34(1.29, 1.4) | 1.3(1.25, 1.35) | 229.01 | 0.38(0.33) | 1.3(1.26) |
| general disorders and administration site conditions | 5482 | 1.3(1.26, 1.33) | 1.23(1.21, 1.25) | 291.12 | 0.3(0.26) | 1.23(1.2) |
| ear and labyrinth disorders | 125 | 1.15(0.97, 1.37) | 1.15(0.96, 1.37) | 2.48 | 0.2(-0.05) | 1.15(0.99) |
| nervous system disorders | 2288 | 1(0.95, 1.04) | 1(0.96, 1.04) | 0.04 | -0.01(-0.07) | 1(0.96) |
| gastrointestinal disorders | 2012 | 0.91(0.87, 0.95) | 0.91(0.88, 0.95) | 17.69 | -0.13(-0.19) | 0.91(0.88) |
| renal and urinary disorders | 585 | 0.89(0.82, 0.97) | 0.89(0.82, 0.96) | 7.41 | -0.16(-0.28) | 0.89(0.84) |
| infections and infestations | 1288 | 0.89(0.84, 0.94) | 0.9(0.85, 0.95) | 16.41 | -0.16(-0.24) | 0.9(0.86) |
| respiratory, thoracic and mediastinal disorders | 1207 | 0.88(0.83, 0.93) | 0.88(0.83, 0.93) | 19.38 | -0.18(-0.26) | 0.88(0.84) |
| investigations | 1659 | 0.86(0.81, 0.9) | 0.86(0.83, 0.89) | 37.94 | -0.21(-0.28) | 0.86(0.83) |
| cardiac disorders | 803 | 0.83(0.77, 0.89) | 0.84(0.79, 0.89) | 27.14 | -0.26(-0.36) | 0.84(0.79) |
| vascular disorders | 477 | 0.71(0.65, 0.78) | 0.72(0.65, 0.79) | 53.67 | -0.48(-0.61) | 0.72(0.67) |
| metabolism and nutrition disorders | 480 | 0.69(0.63, 0.76) | 0.7(0.63, 0.77) | 65.12 | -0.52(-0.65) | 0.7(0.65) |
| neoplasms benign, malignant and unspecified (incl cysts and polyps) | 634 | 0.68(0.63, 0.74) | 0.69(0.64, 0.75) | 90.54 | -0.53(-0.65) | 0.69(0.65) |
| skin and subcutaneous tissue disorders | 756 | 0.58(0.54, 0.63) | 0.6(0.55, 0.65) | 217.77 | -0.75(-0.85) | 0.6(0.56) |
| eye disorders | 264 | 0.55(0.48, 0.62) | 0.55(0.49, 0.62) | 98.33 | -0.86(-1.03) | 0.55(0.5) |
| endocrine disorders | 36 | 0.52(0.38, 0.72) | 0.52(0.37, 0.73) | 15.73 | -0.94(-1.4) | 0.52(0.4) |
| reproductive system and breast disorders | 104 | 0.52(0.43, 0.63) | 0.52(0.43, 0.63) | 46.44 | -0.94(-1.22) | 0.52(0.44) |
| hepatobiliary disorders | 122 | 0.42(0.35, 0.51) | 0.43(0.36, 0.51) | 95.04 | -1.23(-1.48) | 0.43(0.37) |
| psychiatric disorders | 777 | 0.42(0.39, 0.45) | 0.44(0.41, 0.48) | 600.46 | -1.19(-1.29) | 0.44(0.41) |
| immune system disorders | 80 | 0.33(0.27, 0.41) | 0.33(0.27, 0.41) | 107.66 | -1.58(-1.9) | 0.33(0.28) |
| blood and lymphatic system disorders | 167 | 0.3(0.26, 0.35) | 0.31(0.27, 0.36) | 267.14 | -1.7(-1.92) | 0.31(0.27) |
| congenital, familial and genetic disorders | 20 | 0.21(0.14, 0.33) | 0.21(0.14, 0.32) | 57.63 | -2.22(-2.84) | 0.21(0.15) |
